# Anti-Inflammatory and Antioxidant Effects of Carvacrol on *N*-Methyl-*N*′-Nitro-*N*-Nitrosoguanidine (MNNG) Induced Gastric Carcinogenesis in Wistar Rats

**DOI:** 10.3390/nu14142848

**Published:** 2022-07-12

**Authors:** Ayse Gunes-Bayir, Eray Metin Guler, Mehmet Gultekin Bilgin, Ilyas Samet Ergun, Abdurrahim Kocyigit, Agnes Dadak

**Affiliations:** 1 Department of Nutrition and Dietetics, Faculty of Health Sciences, Bezmialem Vakif University, Istanbul 34065, Turkey; mgbilgin@bezmialem.edu.tr; 2 Department of Medical Biochemistry, Faculty of Medicine, University of Health Sciences, Istanbul 34668, Turkey; eraymetin.guler@sbu.edu.tr; 3 Department of Pathology Laboratory, Vocational School for Health Services, Bezmialem Vakif University, Istanbul 34265, Turkey; iergun@bezmialem.edu.tr; 4 Department of Medical Biochemistry, Faculty of Medicine, Bezmialem Vakif University, Istanbul 34093, Turkey; akocyigit@bezmialem.edu.tr; 5 Institute of Pharmacology and Toxicology, Clinical Pharmacology, University of Veterinary Medicine Vienna, 1210 Vienna, Austria; agnes.dadak@vetmeduni.ac.at

**Keywords:** gastric cancer, carvacrol, MNNG, anti-inflammatory, antioxidant, Wistar rat

## Abstract

Carvacrol is a dietary polyphenol from *Lamiaceae* plants that has been shown to possess a wide range of biological activities including antioxidant and antitumor effects. This study aimed to investigate its anti-inflammatory and antioxidant effects on *N*-methyl-*N*′-nitro-*N*-nitrosoguanidine (MNNG) induced gastric carcinogenesis in Wistar rats. Forty-nine rats were randomly assigned to four treatment and three control groups. Over 60 days, MNNG (200 mg/kg BW) was orally applied to animals of groups 1–5 while the rats in groups 2–5 also received different doses of carvacrol (10, 25, 50, and 100 mg/kg BW, respectively) until the end of the experiment. Group 6 rats were treated with 100 mg/kg BW carvacrol and no MNNG whereas group 7 was the control group without any treatment. After the euthanasia of all rats, the inflammatory cytokines and oxidative stress parameters were assessed in the blood and tissues. The expression of caspase 9, Bax, and Bcl-2 proteins in the stomach tissues were investigated through histopathological examinations. Statistically significant differences were observed in the body weight, oxidative stress, and inflammation parameters of groups 1 to 6 compared to group 7 (*p* ≤ 0.001). Animals in MNNG groups 2 and 3 treated with the low dose carvacrol (10 and 25 mg/kg BW) showed significantly reduced oxidative stress, inflammation, and apoptotic effect compared to animals of the MNNG groups receiving increased doses of carvacrol (50 and 100 mg/kg BW) or no carvacrol. Rats exposed to MNNG exhibited gastric cancer cells in several areas. In the MNNG group receiving 100 mg/kg BW carvacrol, the inflammatory cell infiltration was observed in gastric mucosal and submucosal areas whereas MNNG rats supplemented with 10 and 25 mg/kg BW carvacrol showed no pathological alterations of the gastric cells. The results of this study indicate that significant antioxidant and anti-inflammatory effects induced by carvacrol at doses of 10 and 25 mg/kg BW interfered with gastric carcinogenesis induced by MNNG in Wistar rats as well as provide hepatoprotection. However, high doses of carvacrol (50 and 100 mg/kg BW) increased oxidative stress, inflammation, and apoptosis.

## 1. Introduction

Gastric cancer is one of the most common and deadly cancer types in humans, causing more than 700,000 deaths annually [1]. Oxidative stress, increased cell proliferation, angiogenesis, and impaired apoptosis are associated with gastric cancer. The link between inflammation and carcinogenesis is not clearly understood yet [2], but a large number of reports has shown that systematic inflammation plays a significant role in carcinogenesis [3]. Inflammation is a major component of the immune response, which consists of innate and adaptive immunity to protect the human body against many damaging stimuli. Inflammation is the earliest response of the immune system, in which some immune cells, pro-inflammatory cytokines, and chemokines are involved. However, various factors including the inflammatory cell activity, growth factors, activated stroma, and DNA-damage-promoting agents can cause altered cell proliferation and can contribute to the development of structural and functional abnormalities, subsequently leading to cancer development [4]. After trauma-related tissue injury, increased cell proliferation helps in tissue regeneration. In contrast, tumors act as wounds that fail to heal. The inflammatory microenvironment of a tumor comprises a network of numerous immune cells, cytokines, enzymes, and altered signaling pathways. Immune cells can generate or release factors such as tumor growths factor-β (TGF-β), tumor necrosis factor-α (TNF-α), interleukins (IL-1β, IL-6, IL-8, IL-12), interferon-γ (IFN-γ), or reactive oxygen species (ROS) and are capable of establishing a crosstalk network [5,6]. It has been shown that TGF-β contributes to vascularization within the tumor microenvironment by regulating the expression of vascular endothelial growth factor (VEGF) [7]. In addition, TGF-β1 levels have been significantly correlated with poor prognosis for patients with advanced gastric carcinoma [8]. In this context, it is not surprising that the anticancer effects of non-specific anti-inflammatory drugs such as aspirin have been reported [9]. Anti-inflammatory molecules that target key players of the inflammation-to-cancer sequence are of tremendous scientific and therapeutic interest. The modulation of cancer-related inflammation using gene therapies to control or inhibit the growth of cancer cells and improve patient outcome is a growing research area [2]. Nevertheless, the concept of chemoprevention, which was developed decades ago, has also gained increasing scientific interest in recent years [10]. Chemoprevention involves the use of specific natural or synthetic chemical agents that delay, reverse, suppress, or prevent cancer progression from becoming invasive. Therefore, research on the chemopreventive potential of nutritional agents, medicinal plants, and phytochemicals as dietary antioxidants and their ability to induce programmed cell death (apoptosis) has gained momentum [11]. However, to be used clinically, the efficacy and safety of these agents need to be tested in preclinical and clinical studies. In this context, we aimed to elucidate the chemoprotective effects including the apoptotic activity of the *Lamiaceae*-derived monoterpenoid carvacrol in vivo using a MNNG-induced gastric adenocarcinoma model.

Members of the *Lamiaceae* family such as thyme (*Thymus vulgaris*), oregano (*Oregano vulgare*), and sage (*Salvia* spp.) are frequently used in the so-called Mediterranean diet [12]. These plants provide phytochemical diversity and are widely used traditionally for numerous medical purposes. In a study conducted in rats, the effect of *Thymus vulgaris* on antioxidant enzyme activity in various organs was investigated and it was found that *T. vulgaris* consumed as a dietary component reduced the age-related decline in superoxide dismutase enzymes in the liver and heart of aged rats [13]. One active substance representing a significant part of the essential oils of *Lamiaceae* plants is the monoterpenoid phenol carvacrol (5-isoropyl-2-methylphenol) [14,15]. Previously, it has been reported that carvacrol metabolizes fast due to its ring hydroxylation chemical structure, and is excreted after 24 h in the urine of rats [16]. No metabolites were found after 48–72 h of carvacrol application. It was also demonstrated that carvacrol was almost completely absorbed in the stomach and proximal small intestine, and its degradation was 29% in the cecum of pigs [17]. Carvacrol has a wide range of biological activities but may have limited application due to low bioavailability [18]. Therefore, improving the physicochemical instability and bioavailability of carvacrol, its loaded nanoparticle, was investigated in in vitro studies that also showed an anticancer effect against lung and colon cancers [18,19].

Phenolic antioxidants such as carvacrol can lose their antioxidant properties at higher concentrations and gain a pro-oxidant status that induces oxidative stress by generating ROS and apoptosis [15]. In addition to having an anti-oxidant effect, carvacrol is one of the most potent anti-inflammatory compounds characterized through a decrease in the TNF-α level, a suppression of cyclooxygenase-2 expression, and an inhibition of nitric oxide production [20]. Anti-carcinogenic and antiplatelet effects of carvacrol have been reported using the Wistar rat model of leiomyosarcoma induced by 3,4-benzopyren [21]. A study on hepatocellular carcinoma induced by diethylnitrosamine in rats showed that carvacrol supplementation protected the antioxidant system of the rats and prevented lipid peroxidation as well as hepatic cell damage [22]. It has also been found that carvacrol has an anticancer effect by inhibiting cell proliferation and preventing metastasis [23]. Carvacrol in D-galactosamine induced liver injury in rats expressed significant hepatoprotective and antioxidant effects comparable with the standard drug, silymarin [24]. Furthermore, the chemopreventive effects of carvacrol have be seen in a study using a rat model of 1,2-dimethylhydrazine-induced colon carcinogenesis [25]. All in vivo reports have shown the anticancer and antiproliferative effects of carvacrol against different carcinomas except gastric cancer. An in vitro study on human gastric adenocarcinoma cells demonstrated the reactive oxygen species mediated-apoptotic effects of carvacrol at low doses [26]. The same study revealed that carvacrol at a high dose (100 mg/kg BW) could cause pro-oxidative effects. Additionally, the high dose was orally applied to healthy Wistar rats and the treated animals developed higher oxidative stress levels in their stomach tissues and blood compared to the control group rats. An increase in body weight was determined, which was suggested to be caused by the pathological alterations observed in the histopathological examination of stomach tissues of the carvacrol treated rats.

Nitrosamines, as potential carcinogens, are suggested to be risk factors in the human diet that may produce cancer in diverse organs and tissues including the gastrointestinal tract. Exposure to *N*-nitroso compounds such as *N*-methyl-*N*-nitrosourea, MNNG, and their precursors is common in our daily lives and reaches the individual not only through food, but also through a variety of industrial, agricultural, and consumer items such as tobacco products, cosmetics, pharmaceutical products, and agricultural chemicals [22]. Researchers have suggested that long-term exposure to *N*-nitroso compounds may contribute to gastric carcinogenesis [27]. *N*-methyl-*N*-nitro-*N*-nitrosoguanidine (MNNG) is a chemical proven to act as a gastric carcinogen in several animal species and hence is widely used to establish animal models of gastric cancer. MNNG can induce gastric adenocarcinoma without metastasis [28]. The Wistar rat MNNG-model has been accepted as an ideal model for experimental studies to evaluate the biological activity of different compounds in the context of human gastric carcinogenesis [10]. To date, nutritional agents such as curcumin, eugenol, folic acid, genistein, lycopene, naringenin, and tea polyphenols have been studied in gastric adenocarcinoma models induced by MNNG [29,30,31,32,33,34].

Considering the dose-dependent antioxidant and/or pro-oxidant effects of carvacrol described above, in vivo studies on the impact of carvacrol supplementation in the context of gastric carcinogenesis are required. Therefore, this study aimed to investigate the anti-inflammatory and antioxidative effects of carvacrol in a Wistar rat MNNG-model.

## 2. Materials and Methods

### 2.1. Animals

A total of 49 male Wistar rats (Bezmialem Vakif University, Animal Research Center, Istanbul, Turkey), 7–8 weeks of age, were housed for 60 days in plastic cages on hardwood bedding in an air-conditioned biohazard room at a controlled temperature (20 °C to 22 °C) and humidity (30% to 50%) with a 12-h/12-h light-dark cycle. Rats were fed with standard laboratory feed and water ad libitum. The animals were cared for in accordance with national and international guidelines. All procedures were approved by the Ethical Animal Experimentation Committee of the Bezmialem Vakif University under the license number 2016/88.

### 2.2. Sources

For each Wistar rat, the individual MNNG (Tokyo Chemical Industry, Tokyo, Japan) doses (200 mg/kg BW) were weighed under sterile conditions using a precision scale (Ohaus Co., Parsippany, NJ, USA) and kept in a cool, dark place. Each dose was diluted with tap water right before application. The MNNG solution was given from a tube (Eppendorf, Hamburg, Germany) covered with aluminum foil to prevent MNNG photolysis. Carvacrol (Sigma Chemical Co., St. Louis, MO, USA) was weighed sterile using a precision scale (Ohaus Co., Parsippany, NJ, USA) and kept in a cool and dark place to protect against photolysis. Solutions were prepared with tap water on every application day.

### 2.3. Experimental Design

All rats received human care in compliance with the guidelines for the protection of animals used for scientific purposes (Directive, 2010/63 EU, Decision, 2012/707/UE, and RD 53/2013). The animals were randomly assigned to seven groups of seven rats each (Table 1). Rats in group 1 were exposed to MNNG (200 mg/kg BW) dissolved in water by oral gavage application at 10-day intervals [35]. Rats in experimental groups 2–5 were exposed to MNNG (200 mg/kg BW) like the group 1 rats and different doses of carvacrol (10, 25, 50, and 100 mg/kg BW, respectively) per oral gavage three times a week until termination of the experiment. In the present study, the maximum amount of carvacrol given to the rats corresponded to 0.1% of their daily diets [36]. Animals in group 6 received no MNNG doses but the highest dose of carvacrol (100 mg/kg BW) three times a week while animals of group 7 were kept as the control group without treatment.

The body weight of rats was assessed every 10 days until the end of 60 days. After fasting overnight, all animals were sacrificed by cervical dislocation after anesthesia with ketamine/xylazine 35–50/5–10 mg/kg BW.

### 2.4. Necropsy and Samples

All rats were macroscopically examined. Blood samples were collected by intracardiac injection before necropsy. Stomach and liver tissues were collected for pathological, biochemical, and histopathological examinations.

### 2.5. Pathological Examinations

The internal organs were macroscopically examined. The stomach and liver of each animal were washed with ice-cold saline and processed according to the respective protocols of the various examinations and assays conducted. In addition, the proximal duodenum of rats was removed to detect possible carcinogenesis spread [37].

### 2.6. Histopathological Examinations

The collected tissues were fixed in 10% formalin, embedded in paraffin, sectioned, and mounted on poly-L-lysine-coated glass slides. One section from each specimen was stained with hematoxylin and eosin, and one for the Alcian Blue/Periodic Acid-Schiff (AB/PAS). The remaining tissues were used for immunohistochemistry (IHC) staining.

Hematoxylin and eosin (HE) stained 4 μm-sections were assessed from each stomach and liver block to estimate histopathological changes at different magnification factors [38] using a Nikon Eclipse Ci microscope, and NIS Elements Basic Research Microscope Imaging Software (Nikon Instruments Inc., Tokyo, Japan). In order to perform further analysis on the liver tissues, the evaluation of mucins, mucin-like molecules, and other carbohydrates containing macromolecules were detected by AB/PAS staining [39], a method used for the differential staining of glycoproteins. A further analysis of the stomach tissues was performed by IHC staining, which is a highly sensitive and specific detection method for antigens in tissue sections as a result of immunological and chemical reactions [40]. Apoptosis induced by carvacrol via extrinsic and intrinsic pathways has been reported for various cancer cell lines [14]. Therefore, IHC staining was carried out with antibodies against Bax, Bcl-2, and caspase 9 in sections [32]. The high expression of Bax protein helps in the downregulation of the Bcl-2 protein, which may enhance the permeability of the mitochondrial membrane, thereby activating caspase 9 [41]. The anti ß-actin antibody was used as the control. The precise procedures used for IHC staining were as previously described [26]. Briefly, 4 μm-thick sections were deparaffinized and rehydrated through a graded series of ethanol. The retrieval of tissue antigen was conducted using a microwave oven. After the inhibition of endogenous peroxidase activity by immersion in 3% H_2_O_2_ block solution (SensiTek HRP Anti-Polyvalent Staining System, Scytek Laboratories, Inc, Logan, UT, USA), the sections were incubated with the primary antibody and washed thoroughly in PBS. Steps for the incubation of sections were performed at room temperature and slides were rinsed thoroughly with PBS between each step. Glasses were incubated with SensiTek HRP Anti-Polyvalent biotinylated secondary antibody (SensiTek HRP Anti-Polyvalent (DAB) Staining System, Scytek Laboratories Inc., Logan, UT, USA) followed by a streptavidin peroxidase complex (SensiTek HRP, Scytek Laboratories Inc., Logan, UT, USA) and washed with PBS. Finally, immune complexes were visualized by incubation with 0.01% H_2_O_2_ and 0.05% 3,3-diaminobenzidine tetrachloride (DAB Chromogen Substrate Kit, Scytek Laboratories, Inc, Logan, UT, USA). Slides were evaluated according to the staining intensity [42,43,44]. Immunopositive stained cells were seen as brown. The intensity of immunoreactivity was assessed as follows: no staining (0), weak (+1), moderate (+2), and strong (+3) [42,44].

### 2.7. Biochemical Examinations

Serum was obtained from blood samples at 1500× *g* centrifugation for 30 min. The stomach and liver tissues were homogenized at 1500× *g*. The separated plasma and tissue homogenates were used for biochemical analyses.

### 2.8. Inflammation Parameters

Several inflammation markers in serum have been shown to be associated with gastric cancer progress [45,46,47,48]. Therefore, the IL-6, IL-1β, TNF-α, VEGF, and TGF-β levels were determined by photometric methods with commercially purchased ELISA kits in the present study [49].

### 2.9. Oxidative Stress Assay

Oxidative stress plays an important role in the initiation and progression of several diseases such as cancer, cardiovascular disorders, diabetes, and neurological diseases [32]. Oxidative stress can also occur as a result of the reduced capacity of intracellular antioxidant systems. A simple assay can be used for the determination of the total antioxidant and oxidant activities in plasma/serum and other body fluids and their responses to dietary intervention and nutritional supplementation [50,51]. The effect of carvacrol on the oxidant–antioxidant balance in MNNG exposed rats can be determined via intracellular and extracellular oxidative status. Therefore, the total oxidant status (TOS) and total antioxidant status (TAS) were analyzed by the spectrophotometric method and determined using an automated colorimetric measurement method as previously described [48]. The TAS was measured using a new-generation, stable, and colored 2,2-azinobis-(3-ethylbenzothiazoline- 6-sulfonic acid) radical cation (ABTS•+). This is decolorized by antioxidants according to their concentrations and antioxidant capacity and this change in color was measured as a change in the absorbance at 660 nm. This process was performed on an automated analyzer and the assay was calibrated with Trolox. The results were described as mmol Trolox equivalent/L. The TOS was measured using an automated method that allowed the oxidants in the sample to oxidize the ferrous ion-o-dianisidine complex into a ferric ion. The ferric ion formed a colored complex with xylenol orange in an acidic medium. The color intensity was measured spectrophotometrically, which is related to the total quantity of oxidant molecules present. The assay was calibrated with hydrogen peroxide and the results were expressed as mmol hydrogen peroxide equivalent/L. The OSI values were calculated using the TOS/TAS formula (OSI = TOS/TAS).

### 2.10. Statistical Analysis

Power analysis was conducted when planning the present research protocol [35,52]; 95% confidence level and 80% power were calculated for the number of Wistar rats to be used in the experiment. It was considered that gastric adenocarcinoma induced by MNNG would cause losses in these rats or that cancer could not occur in 100% of the rats in the groups receiving MNNG [53,54]. The distribution of the data was examined by the Shapiro–Wilk test. A parametric, one-way ANOVA test was utilized for the comparison of three or more groups with a normal distribution of variables. A non-parametric test, the Kruskal–Wallis test, was used for the comparison of three or more groups with non-normal distributed data. For post hoc analyses of data, Bonferroni and Dunn tests were used to compare the significant variables, respectively. Descriptive statistics of the data are given as mean ± standard deviation and median values. All statistical data were analyzed and reported at α = 0.05 significance level using the Statistical Package for the Social Sciences version 22.0 (IBM Corp., Armonk, NY, USA).

## 3. Results and Discussion

Plant species of the *Lamiaceae* family are rich in phenolic compounds and are mainly used as dried products for culinary purposes and herbal medicine [55]. Carvacrol, as a major component of these plants, has gained great scientific interest [15]. However, the application of carvacrol may be limited due to its volatile nature, low water solubility, and poor bioavailability due to its excretion after 24 h in urine. Therefore, it has been proposed that carvacrol can be incorporated with nanoparticles, liposomes, and emulsions to increase the bioavailability [56]. It has been also reported to have anti-inflammatory, anti-carcinogenic, anti-platelet, chemopreventive, pro-oxidant, antioxidant, antimicrobial, anti-hypernociceptive, hepatoprotective, antispasmodic, antitussive, and anti-obesity activities [14]. The anti-inflammatory and antioxidant effects of different doses of carvacrol applied to Wistar rats in a MNNG-carcinogenesis-model over a period of 60 days were investigated. To our knowledge, this is the first in vivo study describing the impact of MNNG and carvacrol exposure on the organs, tissues, and blood on a macroscopic, microscopic, and molecular level.

### 3.1. Carvacrol Treatment Changed Body Weight of Wistar Rats

**Figure 1:**
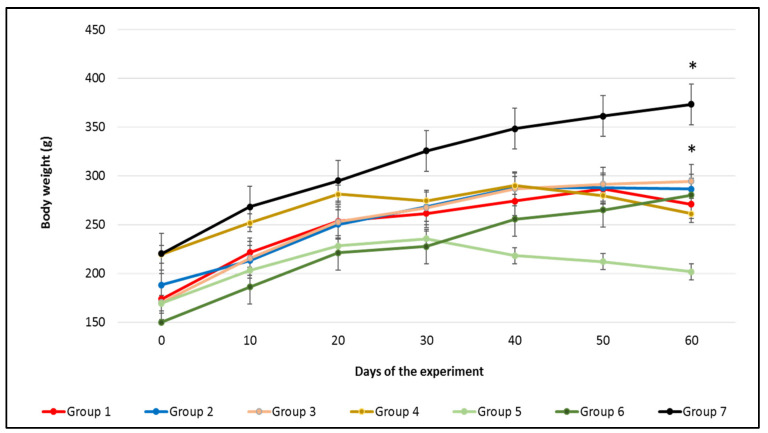
The effect of carvacrol and MNNG application on the body weight of Wistar rats during the experiment. Standard deviations (SDs) are given as mean ± SD. * Values are significantly differed between their initial and final BW at *p* ≤ 0.001.

The body weight (BW) of Wistar rats from all groups were recorded individually in 10-day-intervals to monitor the general well-being of the animals (Figure 1). The final BW of animals in groups 6 (carvacrol 100 mg/kg BW; no MNNG) and 7 (control) significantly differed compared to their initial BW (*p* ≤ 0.001). Changes between the initial and final BW were significantly different in the rats in groups 6 and 7 (*p* ≤ 0.001). Rats in groups 4 and 5 that were exposed to MNNG and high doses of carvacrol (50 and 100 mg/kg BW, respectively) showed a decreased final BW compared to their initial weights. Animals in groups 2 and 3 that were exposed to MNNG and low doses of carvacrol (10 and 25 mg/kg BW, respectively) gained more weight than rats in group 1 (MNNG only). However, differences in the BW changes in groups 1 to 5 were not significant over the course of the experiment. Similarly, rats exposed to diethylnitrosamine and pre-/post-treated with carvacrol (15 mg/kg BW) showed an increase in the final BW when compared to their initial BW [22]. However, there was a significant decrease in the final BW of rats exposed to the hepatocarcinogen diethylnitrosamine when compared to the control group rats. In our study, the BW measurement from the carcinogen exposed rats (group 1) as well as rats exposed to MNNG with 50 mg/kg BW carvacrol (group 4) and with 100 mg/kg BW carvacrol (group 5) showed a decrease at 60 days in comparison to the rats in groups 6 and 7.

### 3.2. Body, Organs and Tissues of Wistar Rats Affected by Carvacrol and MNNG Exposure

At necropsy, careful observation and dissection of the bodies and organs were carried out as well as samples collected for additional examination. Group 7 rats macroscopically revealed no pathological changes in the organs or tissues, whereas rats from groups 1 to 6 showed tense abdomen and bowels with gas formation (Figure S1). Livers from the group 1 rats were swollen, while swelling of the stomach was observed in rats from groups 4, 5, and 6. There was no macroscopically visible alteration in the stomach tissues in the group 7 rats. Hyperemic stomach tissues with poor elasticity and fine wrinkles were found in rats from groups 1, 4, and 6. Tissues from groups 4 and 6 were rubor and thinned. Thickening of the stomach wall was seen in the group 1 rats. A recent study on MNNG-induced gastric carcinogenesis in rats described the gastric mucosa with poor elasticity, little mucus, and fine wrinkles [57]. In addition, the proximal duodenum of these rats was examined, revealing that the application of MNNG (100 micrograms/mL) for 16 weeks (112 days) resulted in tumor incidences of the proximal duodenum [37]. The present study was conducted over a period of 60 days, and duodenal tissues obtained from the animals in MNNG groups 1 to 5 did not show any changes compared to the rats receiving no MNNG (groups 6 and 7).

**Figure 2:**
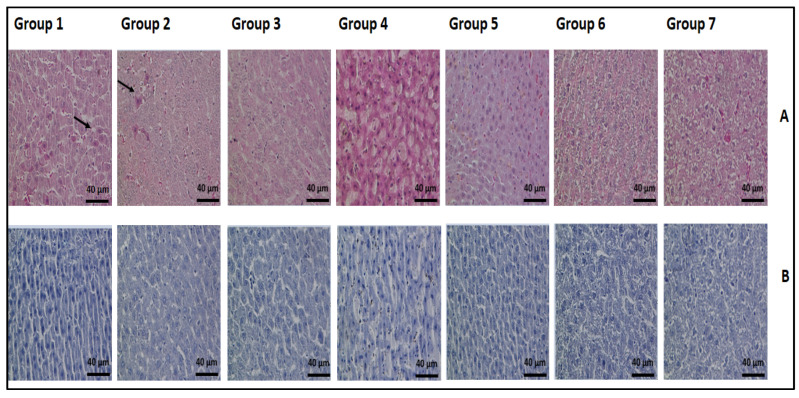
The histopathological examination of the liver tissue in all groups of rats (magnification × 400). (**A**) H&E-stained sections; (**B**) AB/PAS-stained sections. Arrows point to the indication area.

Histopathological examinations of the representative liver tissues from all groups were carried out by H&E and AB/PAS staining (Figure 2A,B). The assessment of stained tissues was performed at different magnification factors to estimate the histopathological changes. No pathological alterations in the livers from the rats in groups 3, 5, 6, and 7 were observed. The group 1 rats presented normal hepatocytes, Kupffer cells, and numerous acidophil (apoptotic) bodies, which presented as hypereosinophilic cytoplasm. Additionally, the group 2 rats had acidophil bodies, but were less common than the group 1 rats. The morphological assessment is still the “gold standard” in the identification of apoptotic cell death [58]. During the apoptosis of cells, they shrink and lose contact with their neighboring cells. The condensation of chromosomes and the fragmentation of cytoplasm and nucleus result in membrane-bound subcellular fragments that are called apoptotic bodies. Group 4 (50 mg/kg BW carvacrol) rats showed focal necrosis of hepatocytes with inflammation while all other groups were without steatosis, inflammation, fibrosis, and necrosis. In our study, high doses (100 mg/kg BW) and low doses (10 and 25 mg/kg BW) of carvacrol resulted in hepatoprotective effects against MNNG application in rats. Likewise, the oral application of carvacrol (80 mg/kg BW) for 21 days showed a hepatoprotective effect in D-galactosamine (D-GaIN) induced liver injury in rats [23]. Rats pretreated with carvacrol (15 mg/kg BW) one week before the administration of diethylnitrosamine with a duration of 16 weeks were also protected against hepatocellular carcinogen [22]. It was suggested that the mechanism of its hepatoprotective effects is related to its protection against the structural integrity of the hepatocellular membrane and the pro-oxidant/antioxidant balance in the liver.

**Figure 3:**
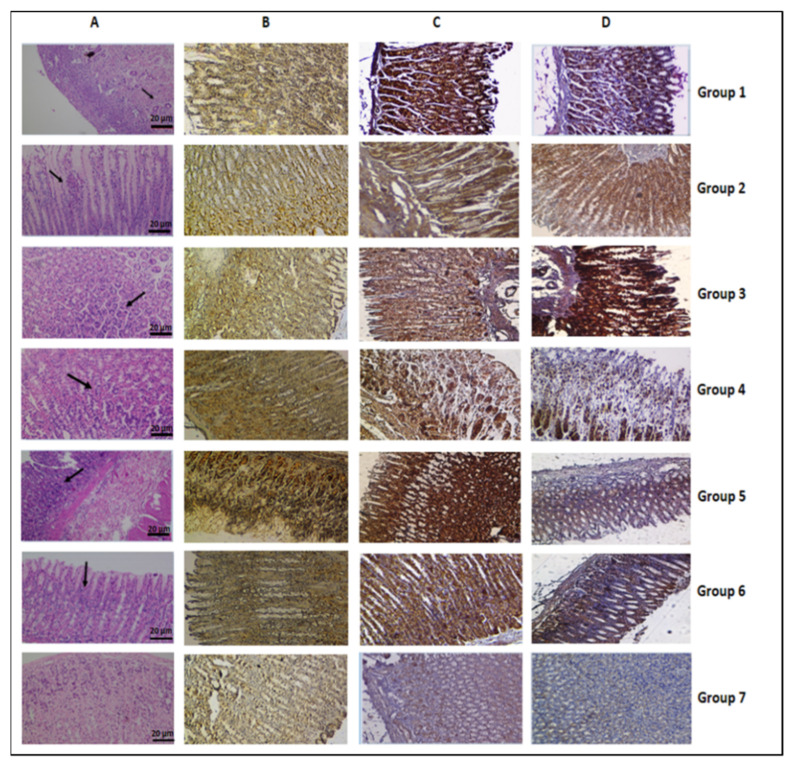
The histopathological examination of stomach tissue in all groups of rats (magnification × 200). (**A**) H&E-stained sections; IHC-stained sections for the caspase 9 (**B**), Bax (**C**), and Bcl-2 (**D**) proteins (magnification × 200), respectively.

In the present study, MNNG was used to establish gastric carcinogenesis in vivo. The stomach tissue of rats exposed to MNNG (group 1) exhibited gastric cancer cells in several areas (Figure 3A), indicating the successful establishment of the MNNG-induced animal model. Stomach tissues from all groups stained with H&E and IHC are shown in Figure 3A–D. The assessment of stained tissues was undertaken at different magnifications. The gastric mucosa of the group 1 rats was atrophic and thin, the glands were significantly reduced, and the arrangement was irregular. Similar observations were reported in a study investigating the epigallocatechin gallate effects on MNNG-induced gastric carcinogenesis in rats [59]. Inflammatory cell infiltration in gastric mucosal and submucosal areas were observed in tissue from rats in group 5 that were exposed to MNNG and a high dose of carvacrol (100 mg/kg BW). Tissues from groups 2, 3, 4, and 7 presented normal gastric cells in the oxyntic mucosa of the gastric corpus while normal foveolar and parietal cells of gastric mucosa were found for group 6 rats exposed to only a high dose of carvacrol.

A large number of in vitro studies have reported that carvacrol showed anticarcinogenic potential through its cytotoxic, apoptotic, and genotoxic effects [14]. Apoptosis as well as apoptosis-associated alteration play an important role in carcinogenesis and tumor development [60]. Apoptosis may occur due to two different pathways such as the extracellular (extrinsic) death receptor and intracellular (intrinsic) mitochondrial pathways, requiring the activation of the caspase-cascade. A key role as an initiator caspase is played caspase 9, which is activated by cytochrome-c release from the mitochondria, and so it activates the effector caspases to introduce the apoptosis of cells [41,61]. In vitro studies on different cell lines have demonstrated that carvacrol induces apoptosis via both pathways in a dose-dependent manner [14]. In particular, the increased activation of Bax, caspase 3, and caspase 9 proteins were determined while decreased levels for Bcl-2 proteins were found. Since pro-apoptotic proteins (Bax) and caspase 9 as well as anti-apoptotic proteins (Bcl-2) are modulators in carcinogenesis [32,41,61], IHC-staining of stomach tissues obtained from all groups was carried out in the present study (Figure 3B–D). Slides were scored based on the intensity of the brown color [42,43,44], and a comparison of the IHC stained slides from the group 7 rat tissues. Assessments by IHC-staining revealed a low expression level for caspase 9, Bax, and Bcl-2 proteins in gastric tissues from the group 7 rats (control) compared to the other groups. MNNG-exposed rats (groups 1) and rats exposed to MNNG and 50 mg/kg BW carvacrol (group 4) revealed the same levels for the caspase 9 and Bcl-2 proteins while the Bax level in group 1 was less high in gastric cells. Caspase 9 and Bax protein expressions were the lowest in the group 2 and 3 rats, and the Bcl-2 protein expression level for these groups was high in comparison to all of the other groups. Group 3 rats showed higher Bcl-2 protein expression level while the caspase 9 and Bax protein expression levels were lower compared to group 2 (MNNG and 10 mg/kg BW carvacrol). The expression levels (mg/kg carvacrol BW) of caspase 9 and Bax proteins were highest in the group 5 rats (100 mg/kg BW carvacrol and MNNG) compared to all of the other groups. On the other hand, the results from group 6 (only 100 regarding caspase 9, Bax, and Bcl-2 protein expression levels) resembled the results from group 4 (Figure 3). Similar to a previous study [25], this apoptotic activity at high doses (50 and 100 mg/kg BW) may be based on the pro-oxidant character of carvacrol. In another study, the administration of high doses of eugenol (100 mg/kg BW) with MNNG (150 mg/kg BW) decreased the Bcl-2 protein expression level and increased the expression of the Bax and caspase 9 proteins compared to only the MNNG-exposed rats and control group [32]. MNNG is a carcinogenic substance that can induce DNA damage and increase mutations and genotoxicity [10,28,29,30,31,32,33]. Cells exposed to carcinogens can undergo apoptosis or escape from apoptosis. In this case, MNNG can induce apoptosis due to the increased caspase 9 and Bax protein expressions and decreased Bcl-2 protein level. An upregulation of Bcl-2 and downregulation of Bax, caspase 9, and caspase 3 were also observed in the MNNG (150 mg/kg BW) administered rats [32]. Cells escaping apoptosis develop cancer by increasing or decreasing the expression of anti- or pro-apoptotic proteins. In the present study, low doses of carvacrol given to rats in groups 2 (10 mg/kg BW) and 3 (25 mg/kg BW) may prevent the toxic effect of MNNG through the low expression of caspase 9 and Bax, and the high expression of the Bcl-2 protein levels. In addition, a large number of in vitro studies have reported that carvacrol can induce DNA damage, caspase activation, PARP cleavage, ROS generation, and Bcl-2 gene expression, therefore leading to apoptosis in cancer cells [15]. Therefore, low doses of carvacrol (10 and 25 mg/kg BW) exposed rats were prevented from MNNG-induced gastric adenocarcinogenesis while those exposed to high doses (50 and 100 mg/kg BW) of carvacrol exhibited synergistic activity causing apoptosis. However, biochemical analysis was performed to determine which dose of carvacrol showed better protection against GC.

### 3.3. Carvacrol Dose-Dependently Reduces Inflammation in MNNG-Exposed Rats

A link between inflammation and cancer has been suggested in pre-clinical, clinical, and epidemiological research studies [2]. Inflammation can promote the malignant transformation of cells and carcinogenesis. An inflammatory microenvironment contains a large amount of cytokines as well as other inflammatory mediators that impact on immunosuppression, cancer growth, tissue remodeling, and angiogenesis such as TGF-β, VEGF, etc. [2,3].

**Figure 4:**
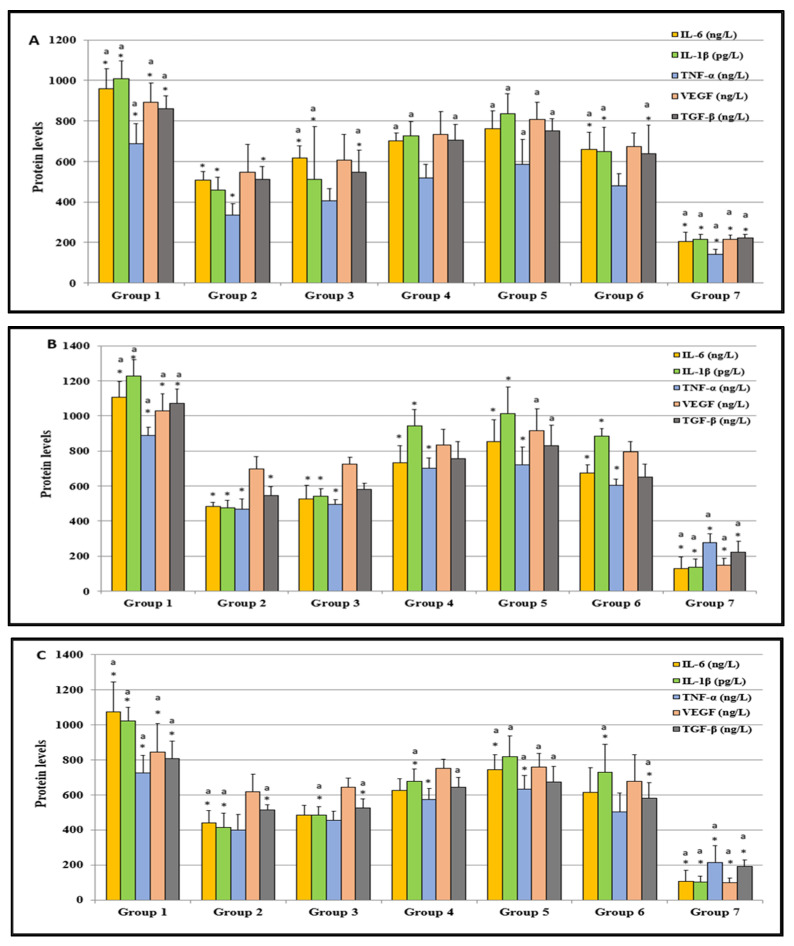
The inflammatory cytokines (IL-1β, IL-6, and TNF-α) and other inflammatory mediators (VEGF and TGF-β) in the serum (**A**), stomach tissues (**B**), and liver tissues (**C**) of all groups were studied. Values are expressed as means ± SD of seven rats (*n* = 7) from each group. * Values differed for the group 1 significantly at *p* ≤ 0.001. ^a^ Values differed for the group 7 (control) significantly at *p* ≤ 0.001.

Figure 4A shows the protein levels of relevant molecules in the serum of Wistar rats at the end of the experiment. The overexpression of TGF-β level is associated with the progression and metastasis of gastric cancer [62]. Many studies have reported that the TGF-β level is related to therapeutic resistance in several cancers. The IL-1β, IL-6, and TGF-β levels were found to be significantly higher in the MNNG-only exposed rats (group 1) compared to the rats in groups 2, 3, 6, and 7 (*p* ≤ 0.001) as well as group 4 (*p* = 0.002). Groups 1, 3, 4, 5, and 6 showed statistically higher levels for IL-1β, IL-6, and TGF-β (*p* ≤ 0.001) compared to group 7 (Figure 4A). The levels of group 2 were less elevated but still significantly higher than the respective levels of group 7 (*p* = 0.016). An association of IL-1β and TNF-α genetic polymorphisms has been found in gastric cancer [63]. Likewise, the TNF-α level of the group 1 rats was statistically significantly increased in comparison to groups 2 and 7 (*p* ≤ 0.001), and group 3 (*p* = 0.024). The TNF-α level of the control group (group 7) was significantly lower than that of groups 1 and 4 to 6 (*p* = 0.001, 0.006, 0.001, and 0.040, respectively). VEGF was found to be critical regarding the invasive process in human gastric cancer [47]. Furthermore, in our study, the VEGF of group 1 showed a significantly higher level in comparison to all of the other groups (*p* ≤ 0.001) (Figure 4A). On the other hand, the VEGF level of the control group was significantly lower compared to all of the other groups (*p* ≤ 0.001). Overall, the results obtained on the inflammation markers in the serum of rats treated with MNNG or MNNG and carvacrol as a potential chemoprotective revealed an inverse carvacrol dose dependent extenuated inflammation, indicating that carvacrol at the lowest dose (10 mg/kg BW) was the most efficient (Figure 4A). Like our results, another study has reported that resveratrol, a non-flavonoid polyphenol, inhibits IL-6 induced invasion of human gastric cancer cells [64].

The IL-1β, IL-6, and TNF-α levels assessed from the stomach tissues of the group 1 rats were statistically significantly higher than those of groups 2 to 7 (*p* ≤ 0.001) (Figure 4B), with the group 7 rats showing the lowest level. The VEGF and TGF-β levels from control group 7 were lower than those of groups 1 and 5 (each *p* ≤ 0.001), and group 4 (*p* = 0.013 and 0.004, respectively). The VEGF and TGF-β levels from group 1 were statistically significantly higher than those in group 2 (*p* = 0.004 and 0.001, respectively) and group 3 (*p* = 0.016 and 0.005, respectively).

The IL-1β and TGF-β levels assessed from the liver tissues of the control group rats were significantly lower than those of groups 1 to 6 (*p* ≤ 0.001) (Figure 4C). The TGF-β levels in the liver tissues of the group 1 rats were found to be significantly higher than the levels of groups 2, 3, 6, 7 (*p* ≤ 0.001), group 4 (*p* = 0.002), and group 5 (*p* = 0.016). The IL-6 and TNF-α levels obtained from rats in the control group were significantly lower than those in groups 1 and 5 (*p* ≤ 0.001). There was no statistical difference for the IL-6 levels among the control group and groups 2 and 3 and for the TNF-α levels between the control group and groups 2, 3, and 6. The IL-6 and TNF-α levels in the liver tissues of group 1 rats demonstrated significant differences compared to groups 2 and 3 (*p* = 0.001, 0.002, 0.004, and 0.033, respectively). The VEGF level measured in the liver tissues of the rats in groups 1, 4, and 5 were significantly higher in comparison to the control rats (group 7) (*p* < 0.001, *p* = 0.002 and 0.001, respectively).

The results for the cytokines (IL-1β, IL-6, and TNF-α) and other investigated inflammatory mediators (VEGF and TGF-β) obtained from the serum and tissue analyses suggest that inflammation in MNNG rats (group 1) can be reduced substantially by supplementation with carvacrol at a low dose (10 mg/kg BW) (group 2). Increasing the carvacrol doses led to the diminishing of effects (groups 3, 4, and 5).

### 3.4. Carvacrol Dose-Dependently Reduces Oxidative Stress in MNNG-Exposed Rats

**Figure 5:**
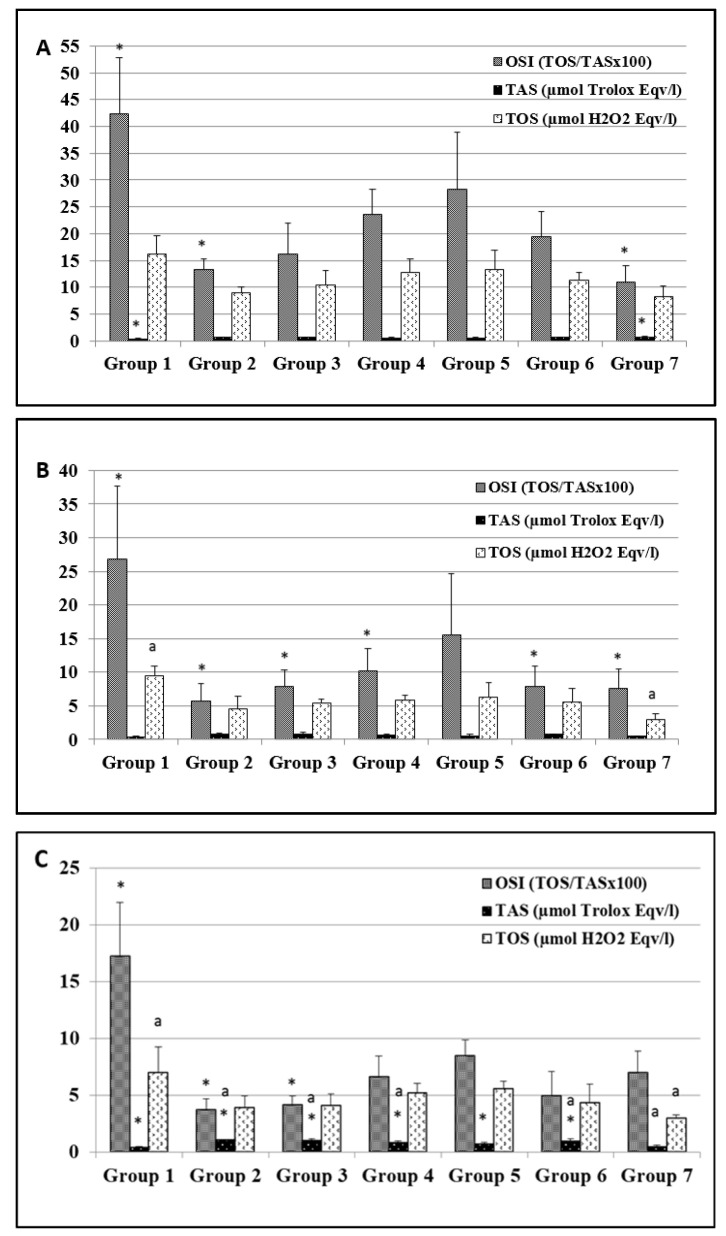
The endogenous oxidative stress markers in the plasma (**A**), stomach tissues (**B**), and liver tissues (**C**) of all groups. Values are expressed as means ± SD of seven rats (*n* = 7) from each group. * Values differed for the group 1 significantly at *p* ≤ 0.001. ^a^ Values differed for the group 7 (control) significantly at *p* ≤ 0.001.

Oxidative stress has a significant impact on humans and may display bad and good qualities for health and/or diseases [60]. Carvacrol supplementation in MNNG-induced carcinogenesis was investigated since oxidative stress can induce both pro-tumorigenic and anti-tumorigenic signaling [65]. The oxidative stress assay was carried out in plasma, stomach, and liver tissues obtained from all rats; their TOS and TAS levels were measured, and the OSI value was calculated (Figure 5A–C). Antioxidant or pro-oxidant effects of carvacrol have previously been reported in in vitro and in vivo studies, except in the gastric cancer model [14,15,21,26]. However, the present study revealed, for the first time, the effects of different doses of carvacrol (10 to 100 mg/kg BW) on MNNG-induced gastric adenocarcinoma.

The OSI value obtained from the plasma analyses of MNNG-only exposed rats (group 1) was significantly higher (Figure 5A) than those in groups 2, 3, and 7 (*p* ≤ 0.001 and 0.017, respectively), while the plasma OSI values obtained from the control group rats was significantly lower than those in groups 4 and 5 (*p* = 0.018 and 0.005, respectively). The TAS levels of the group 1 rats were significantly lower in comparison to groups 2, 3, and 7 (*p* = 0.002, 0.013, and ≤0.001, respectively). The TAS level was significantly higher in the plasma of the control group rats than in the group 5 (*p* = 0.009) animals. The TOS levels were significantly higher in the group 1 rats than in the animals in groups 2 and 7 (*p* = 0.006 and 0.002, respectively).

The OSI values determined in the stomach tissues from the group 1 rats were found to be statistically significant higher in comparison to the other experimental groups, except for group 5 (*p* ≤ 0.001 and *p* = 0.017, respectively), while their TOS levels were higher than in the control group rats (*p* ≤ 0.001) (Figure 5B). The TOS and TAS levels of the group 1 rats were significantly lower than in group 2 (*p* = 0.003 and 0.005, respectively). The TAS and TOS levels of the control group rats were found to be significantly lower compared to groups 2 and 5 (*p* = 0.011 and 0.043).

Changes in the oxidative stress biomarker levels were also investigated in liver tissue. The OSI levels of the group 1 rats were significantly higher compared to groups 2, 3 (*p* ≤ 0.001), and 6 (*p* = 0.004). The TAS levels obtained from the rats in groups 1 and 7 were significantly lower than those in groups 2 to 6 (*p* ≤ 0.001) (Figure 5C). The TOS levels obtained from the group 7 rats were lower in comparison to those in groups 1, 4, and 5 (*p* ≤ 0.001, 0.040 and 0.05, respectively). Similar to the TAS results in our study, the activities of enzymatic antioxidants were significantly decreased in the liver tissue of carcinogen (DMH) exposed rats, whereas the carvacrol (80 mg/kg BW) treated rats showed the same enzymatic antioxidant level as the control group rats [25].

As a result of the endogenous oxidative status in rat tissues, the TOS and OSI levels of the MNNG-exposed rats (group 1) were significantly higher than in all other groups whereas the MNNG-rats supplemented with carvacrol at lower doses (groups 2 and 3) showed the lowest TOS and OSI levels of all MNNG exposed rats (1, 4, and 5) and similar levels compared to groups 6 (100 mg/kg BW carvacrol, no MNNG) and 7 (control). It has been reported that patients suffering from gastric cancer showed high oxidative stress and low antioxidant levels [66]. Our findings correlate with previous reports that suggest that low dose carvacrol possesses antioxidant activities in DEN (diethylnitrosamine)-induced hepatocellular and DMH (1,2-dimethylhydrazine)-induced colon carcinogenesis [16,19]. The authors reported that carvacrol at a dose of 40 mg/kg BW showed a significant chemopreventive effect against DMH-induced colon carcinogenesis in rats. In our study, carvacrol at 10 and 25 mg/kg BW had a beneficial chemopreventive effect on MNNG-induced gastric carcinogenesis.

## 4. Conclusions

This study demonstrated that a low dose of carvacrol has antioxidant and anti-inflammatory properties. From the results, it is evident that carvacrol is capable of modulating apoptosis, significantly decreasing the inflammation process and the endogenous oxidative stress in MNNG-induced gastric adenocarcinogenesis. Furthermore, a decrease in markers for tumor growth and angiogenesis was observed. Based on the significant antioxidant and anti-inflammatory effects induced by carvacrol at doses of 10 and 25 mg/kg BW in MNNG-induced gastric adenocarcinogenesis, it is suggested that carvacrol has the potential to offer beneficial chemopreventive effects in the context of gastric carcinogenesis. In addition, high doses of carvacrol (50 and 100 mg/kg BW) showed pro-oxidant properties that increase oxidative stress, inflammation, and apoptosis. Long-term pre-clinical studies are required, and clinical trials conducted in polluted areas or on smokers who are exposed to nitrosamines would be of special interest in order to determine the clinical potency of carvacrol. Conclusively, more studies should focus on increasing and further evaluating the bioavailability of carvacrol as a chemopreventive agent.

## Figures and Tables

**Table 1:** The study design presents seven groups of Wistar rats (*n* = 7) exposed to MNNG or MNNG and different doses of carvacrol, only high doses of carvacrol, and none of MNNG and carvacrol, respectively.

Groups	MNNG (mg/kg BW)	Carvacrol (mg/kg BW)
1	200	0
2	200	10
3	200	25
4	200	50
5	200	100
6	0	100
7	0	0

## Data Availability

The data presented in this study are available on request from the corresponding author.

## Supplementary Materials

The following supporting information can be downloaded at: https://www.mdpi.com/article/10.3390/nu14142848/s1. Figure S1: Appearance of body (A), organs (B) and tissues (C) from all group representative Wistar rats at necropsy. Size of organs was presented with scale bars.
