# Supplementary material for: Anti-Inflammatory and Antioxidant Effects of Carvacrol on N-Methyl-N′-Nitro-N-Nitrosoguanidine (MNNG) Induced Gastric Carcinogenesis in Wistar Rats
Source: Nutrients. 2022 Jul 12;14(14):2848. doi: 10.3390/nu14142848 (PMC9323991; doi:10.3390/nu14142848)
Supplement: Supplementary file 1 [file nutrients-14-02848-s001.zip › nutrients-1696367-supplementary.pdf]

Supplementary Material

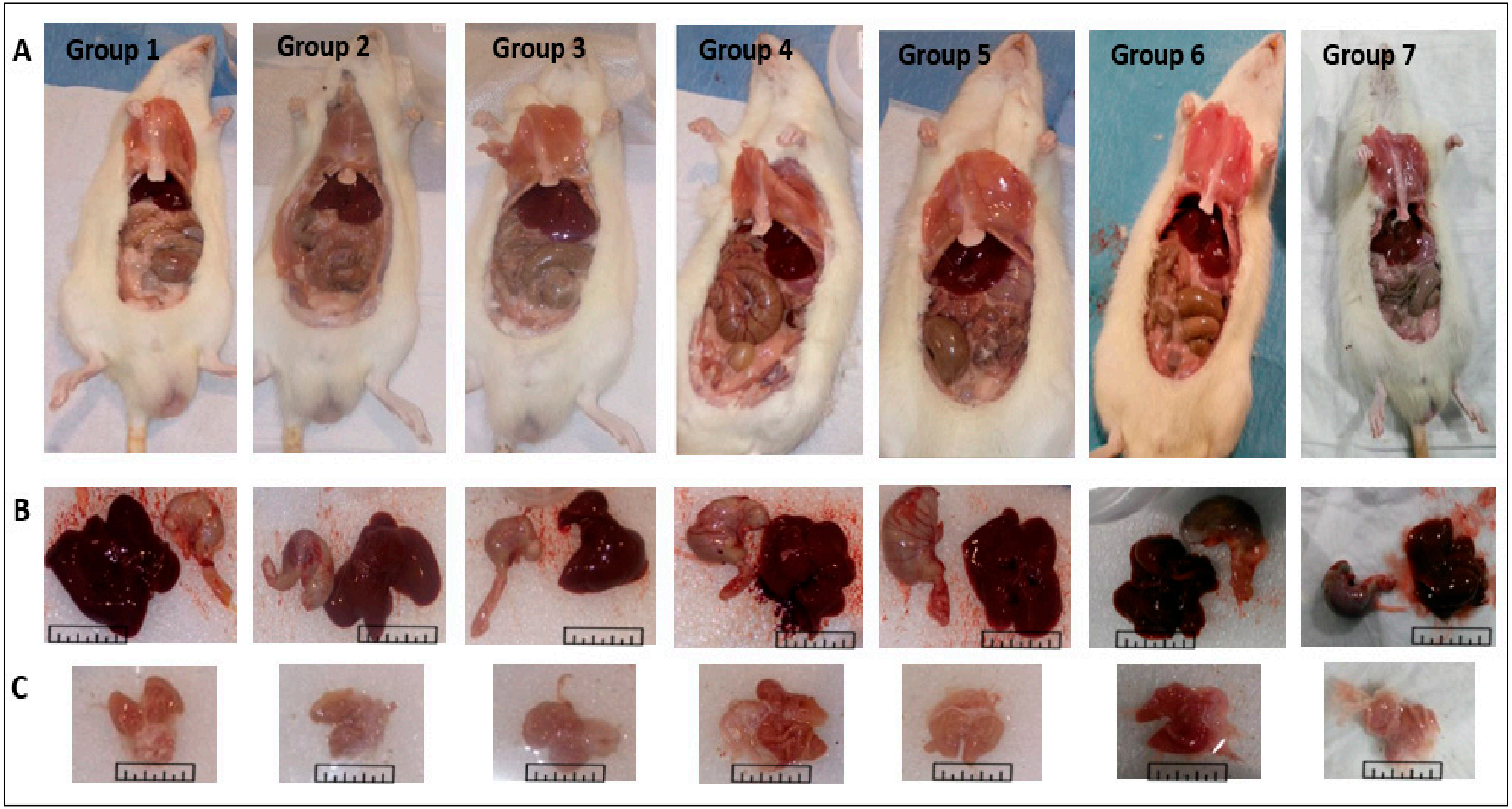

Supplementary Figure S1. Appearance of body (A), organs (B) and tissues (C) from all group representative Wistar rats at necropsy. Size of organs was presented with scale bars.
